# The first articulated skeletons of enigmatic Late Cretaceous billfish-like actinopterygians

**DOI:** 10.1098/rsos.231296

**Published:** 2023-12-06

**Authors:** Tamara El Hossny, Lionel Cavin, Ulrich Kaplan, Achim H. Schwermann, Elias Samankassou, Matt Friedman

**Affiliations:** ^1^ Department of Earth Sciences, University of Geneva, Rue des Maraîchers 13, 1205 Geneva, Switzerland; ^2^ Département de Géologie et de Paléontologie, Muséum d'Histoire Naturelle de la Ville de Genève, CP 6434, 1211 Geneva 6, Switzerland; ^3^ Eichenallee 141, 33332 Gütersloh, Germany; ^4^ LWL-Museum für Naturkunde Westfälisches Landesmuseum mit Planetarium, Sentruper Strasse 285, 48161 Münster, Germany; ^5^ Museum of Paleontology and Department of Earth and Environmental Sciences, University of Michigan, Ann Arbor, MI, USA

**Keywords:** ecological analogue, Cenomanian, billfishes, *Protosphyraena*, Plethodidae, *Rhamphoichthys taxidiotis*

## Abstract

Only few candidates of Mesozoic fishes with a similar body plan and ecological niche to the modern billfishes are suggested as their analogues. Several specimens were recovered from Cenomanian deposits in Germany and Lebanon and display a billfish-like fusiform body with elongated premaxillae. They are found close to the plethodids and show a unique combination of characters (rostrum pointed and extremely elongated, double articular head of the quadrate, anteroposteriorly elongated abdominal centra indicating a slender body and different types of scales on the body) allowing their inclusion in a new genus. Two ‘*Protosphyraena*’ species are also assigned to this new genus. This fish can be considered as an ecological analogue to the extant xiphioids sharing their feeding habits. This fish was abundant and roamed, as an apex predator, the Central Tethys and the Boreal realms during the Cenomanian.

## 1. Introduction

Ray-finned fishes (Actinopterygii) represent about half of all extant vertebrate species and have been the most diverse vertebrates in aquatic environments since the Late Paleozoic. Actinopterygians provide numerous examples of morphological convergence. These patterns are apparent at micro- [1] and macroevolutionary [2] levels. The repeated evolutionary discovery of similar body forms is a recurring theme in history of ray-finned fishes and is often ascribed to shared functional constraints of similar ecological roles [3]. For example, extant ambush predators such as needlefish (e.g. *Belone*) are characterized by an elongated skull, long and thin body and posteriorly placed midline fins; extinct groups like the Permian–Jurassic saurichthyids [4], Jurassic–Cretaceous aspidorhynchiforms [5] and Cretaceous dercetids [6] share similar overall morphologies, although the specific details of their anatomy differ substantially. Billfishes (Xiphioidei; comprising marlins, spearfishes, sailfishes and swordfishes) are another extant group of longirostrine fishes that differ from ambush predators in having a body shape constructed for fast, sustained swimming. Xiphioids have a rich fossil record extending back to the Early Cenozoic [7] but are unknown from Mesozoic deposits. There are few candidates for ecological analogues among fishes of this age, although some Early Jurassic ichthyosaurs may have been billfish-like in their ecology [8,9].

A wealth of Late Cretaceous marine localities yields articulated fossil fishes [10,11]. Complementary preservational styles yield considerable information on fishes of this age, with several closely related species represented by both three-dimensional cranial fossils (e.g. the English Chalk; Woodward [12–22], Friedman *et al.* [23]) and flattened but otherwise complete individuals (e.g. the Lebanese Sanine Limestone; Gayet *et al.* [24]). Despite this wealth of data, body form remains unclear for some of the most enigmatic Late Cretaceous ray-finned fishes: longirostrine tselfatiiforms. Sparse cranial material plus a handful of vertebrae of *Martinichthys* from Coniacian strata of the Smoky Hill Chalk of Kansas, USA, represents the most substantial material of these fishes [25]. These tantalizing specimens, along with rostral fragments from older strata of Cenomanian age in the UK and Italy, suggest a surprising diversity of long-snouted tselfatiiforms. This scant evidence, combined with the variety of body forms among tselfatiiforms, provides few constraints on the overall appearance of these longirostrine fishes [26]. In the absence of more complete anatomical information, it is unclear whether these taxa even represent a single group of tselfatiiforms.

Collecting from the DIMAC Quarry outside of Halle, Westphalia, Germany has yielded a series of articulated pelagic fishes of Late Cenomanian age from the Hesseltal Formation. Arguably the most important discoveries from this locality are multiple articulated specimens of a longirostrine tselfatiiform. These German specimens are joined by a similar longirostrine tselfatiiform from the Lebanese locality of Haqel. Here, we introduce these finds and highlight their implications for the overall morphology of the enigmatic longirostrine tselfatiiforms.

## 2. Geological context and associated fauna

### 2.1. Hesseltal Formation, Germany

The German longirostrine tselfatiiform derives from the Hesseltal Formation in the DIMAC Quarry near Halle, Westphalia. These articulated remains were collected from a narrow (0.44 m) bed identified as the ‘*Chondrites* Event’, just below the plenus bed [27,28]. Due to transpressional tectonics, the macrofossils from the Hesseltal Formation are distorted and stretched in the direction of the striking, as evidenced by the ammonites [29, p. 81, plate 5]. This has affected the fishes described here, and as there was no remark on their original position, this indicates that they should have been distorted, but we cannot estimate the degree and orientation of distortion. These strata fall within the *Metoicoceras geslinianum* Zone and are, therefore, of Late Cenomanian age. The fauna shares several elements with approximately coeval and geographically proximate deposits of the Chalk Group of England [12–23], including the pachycormid *Protosphyraena*, a large pachyrhizodontid, and a tselfatiiform resembling *Dixonanogmius* and an aulopiform similar to *Halec*.

### 2.2. Sannine Limestone Formation, Lebanon

The Lebanese specimen derives from the limestone deposits at Haqel, Lebanon. These deposits have been assigned to different parts of the Cretaceous by previous authors, mainly to the Cenomanian. More recently, Wippich & Lehmann [30] confirmed a Late Cenomanian age for Haqel based on the presence of the ammonite *Allocrioceras cf. annulatum* (Shumard, 1860) [31], which occurs in the lowermost part of the upper Cenomanian *M. geslinianum* Zone (of the international standard). Haqel ‘Fish Shales’ localities [32] have yielded over 70 genera of fish as well as various groups of invertebrates such as molluscs, annelids, crustaceans and echinoderms [33]. This site has recently yielded several tselfatiiforms currently under study.

## 3. Material and methods

### 3.1. Preparation of material

Material from the Hesseltal Formation was prepared mechanically, and the Lebanese material was prepared using a combination of mechanical and chemical techniques, allowing a dorsal view of the skull.

### 3.2. Comparative material

The following material was examined for comparative purposes; the photographs are provided in the electronic supplementary material.

*Martinichthys brevis* KUVP 497 (electronic supplementary material, figure S1); *Martinihcthys ziphioides* KUVP 498 (electronic supplementary material, figure S2); ‘*Protosphyraena*’ *minor* NHMUK P 49758, 32337, 4078 and 49100 (electronic supplementary material, figure S3); ‘*Protosphyraena*’ *stebbingi* NHMUK P 11216 (2) and plaster cast (electronic supplementary material, figure S4); undetermined fossil, MNHN HDJ 250 (electronic supplementary material, figure S5).

### 3.3. Institutional abbreviations

KUVP: University of Kansas Museum of Natural History, Vertebrate Paleontology collection, Lawrence, Kansas, USA; MHNG: Muséum d'histoire naturelle de Genève, Switzerland; MNHN: Muséum National d'Histoire Naturelle, Paris, France; NHMUK: Natural History Museum, London, UK; WMNM: LWL-Museum für Naturkunde, Münster, Germany.

## 4. Results

### 4.1. Systematic palaeontology

Tselfatiiformes Nelson, 1994 [34].

Plethodidae Loomis, 1900 [35].

Genus: *Rhamphoichthys* gen. nov.

**Type species.** *Rhamphoichthys taxidiotis* sp. nov.

**Diagnosis.** Elongate plethotid that differs from all others by the combination of the following characters: fusiform body with a slender rostrum making up half the length of the skull; presence of sclerotic ring; a broad and double-headed articular head of quadrate; hyomandibula hourglass shaped; seven broad branchiostegal rays; vertebral column with at least 100 vertebrae (55 abdominal + 40 caudal); abdominal vertebral centra elongate anteroposteriorly; dorsal fin rays very long with some rays exceeding depth of the body; hypural plate large made of fusion of hypurals 1–4 and an autogenous dorsal hypural 5; scales of different types: weakly mineralized ovoid scales, more robustly mineralized rhombic scales, bilaterally symmetrical scales with midline ridge and notch and at least one scale with a serrated posterior margin.

**Etymology.** From the Greek: *rámfos* (rhamphos) meaning beak with the suffix ichthys for fish.

*Rhamphoichthys taxidiotis* sp. nov.

Zoobank LSID: urn:lsid:zoobank.org:pub:81FE170B-989C-4BD1–8DE1-D73C4EA993B4.

**Holotype.** WMNM P 48342, articulated skull and highly disrupted postcranium.

**Horizon.** Lowermost horizon of upper Cenomanian of the Hesseltal Formation, Halle, Westphalia, Germany.

**Referred material.** Three individuals in various states of completeness and articulation: WMNM P 48344 (electronic supplementary material, figure S6), damaged skull with intact postcranium and WMNM P 64279, incomplete skull and postcranium, both specimens from the lowermost horizon of upper Cenomanian of the Hesseltal Formation, Halle, Westphalia, Germany; MHNG GEPI V5785, complete skull preserved in ventral and dorsal views, with anterior portion of the vertebral column, specimen from the lowermost part of the upper Cenomanian of Sannine Limestone Formation, Haqel, Lebanon.

**Diagnosis.** Same as genus, single species.

**Etymology.** From the Greek *taxidiotis*, meaning traveller. The combination makes it ‘the traveller fish with a beak’, reflecting the long rostrum and wide geographical distribution of this presumably pelagic taxon.

### 4.2. Description

**Figure 1.**
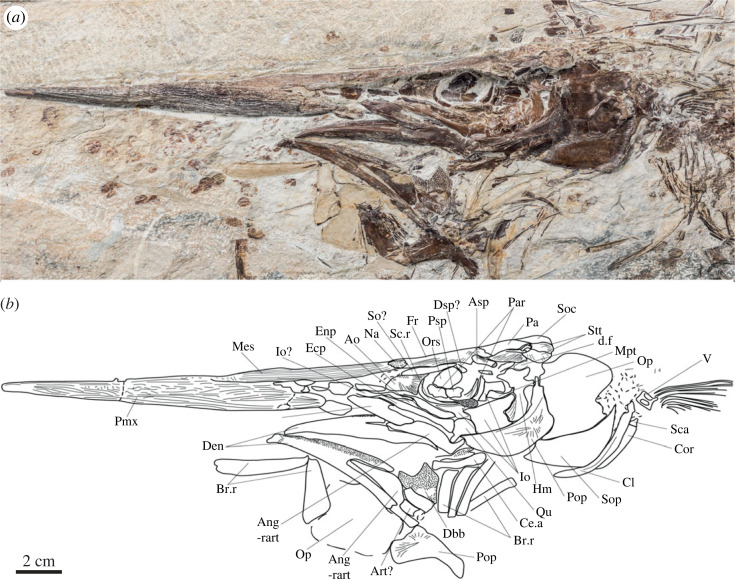
Cranial anatomy of *Rhamphoichthys taxidiotis* gen. et sp. nov., holotype WMNM P 48342. (*a*) Photograph of the skull in left lateral view. (*b*) Interpretive line drawing of (*a*). Ang-rart, angulo-retroarticular; Ao, antorbital; Art, articular; Asp, autosphenotic; Br.r, branchiostegal rays; Ce.a, anterior ceratohyal; Cl, cleithrum; Cor, coracoid; Den, dentary; d.f, dilatator fossa; Dsp, dermosphenotic; Ecp, ectopterygoid; Enp, endopterygoid; Fr, frontal; Hm, hyomandibular; Io, infraorbitals; Mes, mesethmoid; Mpt, metapterygoid; Mx, maxillary; Na, nasal; Op, operculum; Ors, orbitosphenoid; Pa, parietal; Par, parasphenoid; Pmx, premaxillary; Pop, preoperculum; Psp, pterosphenoid; Qu, quadrate; Sc.r, sclerotic ring; Sca, scapula; So, supraorbital; Soc, supraoccipital; Sop, suboperclum; Stt, supratemporal; V, vertebrae.

The description of *R. taxidiotis* is based on the holotype WMNM P 48342 (figure 1) unless stated otherwise.

#### 4.2.1. Neurocranium

**Figure 2.**
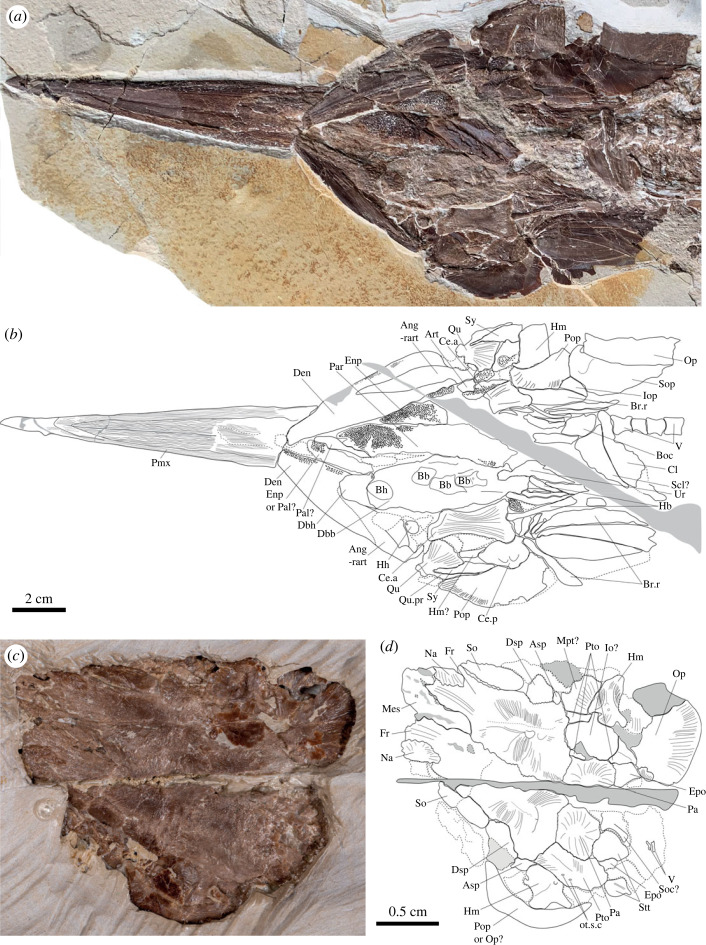
Cranial anatomy of *Rhamphoichthys taxidiotis* gen. et sp. nov., MHNG GEPI V5785. (*a*) Photograph of the skull in ventral view. (*b*) Interpretive line drawing of (*a*). (*c*) Photograph of the skull in dorsal view. (*d*) Interpretive line drawing of (*c*). Ang-rart, angulo-retroarticular; Art, articular; Asp, autosphenotic; Bb, basibranchial; Bh, basihyal; Boc, basioccipital; Br.r, branchiostegal rays; Ce.a, anterior ceratohyal; Ce.p, posterior ceratohyal; Cl, cleithrum; Dbb, dermobasibranchial; Dbh, dermobasihyal; Den, dentary; Dsp, dermosphenotic; Enp, endopterygoid; Epo, epiotic; Fr, frontal; Hb, hypobranchial; Hh, hypohyal; Hm, hyomandibular; Iop, interoperclum; Mes, mesethmoid; Mpt, metapterygoid; Na, nasal; Op, operculum; ot.s.c, otic sensory canal; Pa, parietal; Pal, palatine; Par, parasphenoid; Ph, parahypural; Pmx, premaxillary; Pop, preoperculum; Pro, prootic; Psp, pterosphenoid; Pto, pterotic; Qu, quadrate; Qu.pr, quadratic process; Scl, supracleithrum; So, supraorbital; Soc, supraoccipital; Sop, suboperclum; Stt, supratemporal; Sy, symplectic; V, vertebrae.

The premaxillae form a long rostrum that is suturally integrated with the neurocranium, so these bones are discussed here rather than with the jaws below. The snout makes up roughly half the length of the neurocranium. The rostrum has a pointed to slightly rounded tip and is broken at mid-length in P64279, showing that it is hollow. A conspicuous suture separates the premaxillae along their ventral midlines and intersects the apex of a triangular notch that separates the bones posteriorly. The premaxillae are completely fused along the midline in the anterior quarter of the rostrum. Irregularly anastomosing ridges, roughly aligned with the long axis of the rostrum, ornament all external surface of the premaxillae. In the posterior half of the bone, a longitudinal suture divides the portion of the premaxilla from the mesethmoid, the latter surface of which bears medially directed ridges and grooves. Dorsally, in V5785 (figure 2), a small part of the mesethmoid separates the anterior tips of the frontals. V5785 shows both nasals joining the lateroanterior margin of the frontals. The suture between the nasals and the frontals is formed by interdigitations of the superficial ornamentation of these bones.

The frontal bears a lateral expansion over the orbital region. V5785 shows that each frontal bears a low prominence on the dorsal surface near the centre of ossification. Radial ridges and grooves ornament the middle and posterior parts of the frontal. The lateral margin of the frontal in V5785 exhibits two concavities for the supraorbital anteriorly and the dermosphenotic posteriorly, in addition to the one for the nasal anterolaterally. The frontals join surrounding bones by interdigitating sutures formed by the superficial ridges. Both the supraorbital and dermosphenotic are crushed on the holotype. The parietals display radial ridges and grooves similar to those of the frontals. The preserved portions of the parietals indicate that both ossifications suture along their whole median margin (medioparietal), without separation by the supraoccipital posteriorly. The pterotics lie lateral to the parietals and posterior to the frontals in V5785. The left pterotic is entirely preserved showing a rectangular shape and a slight ornamentation on its surface. An undulating shallow groove marks the path of the otic sensory canal, with two pores visible. The posterior corner of the pterotic forms a rounded process and curves to contact the epiotic. A thick, longitudinal ridge separates the more horizontally oriented mesial region of the pterotic from a more vertically oriented, lateral region of the bone and defines the upper boundary of the dilatator fossa.

In dorsal view, an elongated rectangular autosphenotic protrudes posterolaterally from the skull roof at the level of the suture between the frontal and the pterotic. The lateral wall of the pterotic sutures with a posterior extension of the autosphenotic below the level of the skull roof. Viewed dorsally, V5785 shows small epiotic wedged between the pterotics on its lateral side and the supraoccipital. The epiotic bear a posterior protruding bulge slightly below the level of the skull roof that extends to the level of the pterotic process. The supraoccipital bone is partly visible between the epiotics. It only notches the posteromedian margin of the parietals. The supraoccipital crest is well defined but short and low as seen on the holotype laterally. Two supratemporals are present.

Portions of the parasphenoid are exposed in the orbital region. The ventral surface of the parasphenoid bears a dental plate marked by a series of perforations and with a rounded posterior margin. V5785 shows an elongated parasphenoid with almost the same width over its entire length and with a rounded anterior end bearing a few teeth. The dorsal part of the bone extends as a low, mesially inset lamina suggesting that an interorbital septum formed by the orbitosphenoid and pterosphenoid was present. Portions of this septum are visible in photographs and a cast of the holotype, but the bones appear to have been damaged during moulding. Anterior to the orbit, a splint-like exposure of bone likely represents an extension of the parasphenoid. A bone exposed in the midline of the broken rostrum is either the parasphenoid or the vomer. In ventral view, on V5785, a small square shaped bone with rounded angles could represent the basioccipital.

#### 4.2.2. Circumorbital bones, cheek and sclerotic ossicle

The circumorbital bones are disrupted. A triangular antorbital defines the anterior margin of the orbital opening. It bears a low, curved thickening for the sensory canal and prominent radiating ridges ornament on its posterior half. Both the supraorbital and the dermosphenotic are crushed on the holotype, so they are described from V5785. The paired supraorbitals are elongated and oval shaped located lateral to the frontals, in dorsal view, and ornamented with smooth radiating ridges. The right one is more complete. It extends over the middle third of the frontal on its lateral margin, slightly overlapping it. The dermosphenotics have a distorted circular shape. They bear smooth ornamentation similar to that of the supraorbitals and a similar interdigitating pattern on their margin where they are attached to the frontals. The dermosphenotics and the supraorbitals are tightly sutured to each other and to the frontals.

At least four infraorbitals are present, the anterior ones are thin and small, whereas the posterior ones are larger. It is unclear whether incompletely exposed bone dorsal to the fourth infraorbital is a fifth member of the series, or instead represents displaced scales. Broken fragments of these posterior infraorbitals found posterodorsal to the hyomandibula indicate that they extended far posteriorly, likely partially overlapping the preopercle.

The sclerotic ossicle is crushed and incompletely preserved in all available specimens.

#### 4.2.3. Suspensorium

The palate is long and narrow, with the hyomandibula, ectopterygoid and quadrate being clearly exposed.

A shallow groove appears to mark the suture between the narrow posterior extension of the palatine and the much larger ectopterygoid. The anterior regions of both bones are obscured by the first infraorbital. The ectopterygoid extends from the anterior margin of the antorbital to the posterior half of the orbit. It is rod-like and constricted at mid-length and broadens conspicuously at its posterior junction with the quadrate. The endopterygoid is incompletely exposed, limited to a small area of smooth bone visible dorsomedial to the ectopterygoid. Several fragments of the toothed buccal surfaces of the endopterygoid and ectopterygoid can be seen in a ventral view in V5785.

The hyomandibula is hourglass shaped, with a prominent constriction in its dorsal third. A well-developed opercular process extends from the posterior margin of the hyomandibula at the junction of the ventral and dorsal expansions of the bone. A thickened ridge extends along the lateral surface of the ventral expansion, near its posterior border. The broad articular heads of the hyomandibula join the articular facet on the pterotics. The opening for the hyomandibular branch of the facial nerve (VII) can be seen on this specimen. The metapterygoid joins the anterior margin of the ventral limb of the hyomandibula. Infraorbitals obscure the junction between the metapterygoid and the quadrate, which has a gently curved posterodorsal margin and a well-developed articular condyle that ends from its anteroventral corner. V5785 shows ventrally that the quadrate is fan shaped, with an elongate process extending posteriorly and receiving the symplectic which fits into a notch in the bone. The broad articular condyle of the quadrate bears two heads. The symplectics are triangular, with a broad posterior extremity that tapers ventrally.

#### 4.2.4. Jaws

The maxilla is flat and trapezoidal, lacking any obvious articular process at its anterior end but bearing a well-developed notch at its posterodorsal corner for the supramaxilla. This bone, however, is not preserved.

The lower jaws are intact, with the left mandible exposed in lateral view and the displaced right mandible exposed in mesial view. The lower jaw is slender, with a symphysis that appears somewhat downturned. The dentary constitutes much of the jaw. It bears a ‘V’-shaped notch for the angular posteriorly. A band containing tiny alveoli extends along the dorsal margin of the dentary and is clearest in mesial view. Minuscule, needle-like teeth are preserved in association with some of these alveoli on the mesial side of the dental band of the right mandible. In lateral view, the posteroventral ramus of the dentary appears greatly thickened relative to the posterodorsal ramus and bears strongly developed ornament ridges. The right dentary of V5785 is covered in its middle part by the anterior tip of the dermobasihyal of the lingual plate. The angular inserts into the posterior notch of the dentary. Some ornament ridges radiate from the posteroventral corner of the angular on its lateral face, but these are not as conspicuous as those on the dentary. The angular and retroarticular are fused and together form a broad articular facet for the double-headed condyle of the quadrate in V5785. The articular remains autogenous. A small sediment-filled depression, visible in mesial view behind the posterior edge of the glenoid, might represent the entrance of the mandibular sensory canal.

#### 4.2.5. Opercular series

The opercular series minimally comprises a preopercle, opercle, subopercle and several branchiostegal rays. The preopercle is crescentic, with a smaller dorsal limb that ends in a pointed distal tip and a larger ventral limb that terminates bluntly. A series of fine grooves radiate from the centre of the bone. A thickened band bearing an irregular pattern of small pits extends through the preopercle slightly set off from the anterodorsal margin and marks the position of the preopercular sensory canal. The opercle is semicircular. The subopercle also has a gently rounded posteroventral margin, and it is almost as large as the opercle. A fragment of the interopercle is wedged between a branchiostegal ray and the preopercle in V5785. There are seven branchiostegals seen in V5785, broad and plate-like, rather than rod-like or filamentous.

#### 4.2.6. Branchial arches and ventral hyoid arch

Not all components of the basibranchial series and ventral hyoid arch are visible. A broad plate with a gently convex surface pitted with alveoli represents the fused tooth plates of basibranchials 1–3. It bears well-defined ‘V’-shaped notch along its posterior border. A narrow exposure of bone bearing similar alveoli is apparent immediately ventral to the articulation of the lower jaw, and presumably represents another basibranchial tooth plate. As in the holotype, V5785 shows oval shaped basibranchials complex (1–3) in ventral view, but this is preceded by a dermobasihyal forming an anterior rounded tip for the lingual tooth plate. The latter is ornamented by strong ridges present mainly at the centre. V5785 additionally shows an anterior basihyal and three posterior basibranchials aligned along the long axis of the lingual tooth plate. The anterior ceratohyal is incompletely exposed. It is elongate, slightly curved and bears a longitudinal sulcus on its lateral surface in the holotype. In V5785, the anterior ceratohyal is hourglass shaped and is preceded by a hypohyal, which is pierced by a foramen for the hyoidean artery. The posterior ceratohyal is a triangular bone with rounded corners slightly shifted position and located posterior to the anterior ceratohyal in V5785. It exhibits two well-marked depressions in its posterior part. Several hypobranchial arches, of different lengths and width shorter than the branchiostegal rays, are attached to the posterior part of the basibranchial plate in V5785.

#### 4.2.7. Pectoral girdle and fin

The shoulder girdle is badly damaged and partially concealed by the opercular series. However, it is clear that the cleithrum has a long ventral limb associated with a high placement of the pectoral fin just ventral to the level of the axial column. The coracoid is partially preserved as impression and consists of a slender limb that parallels the ventral limb of the cleithrum. Dorsally, the coracoid joins the scapula, marking the position of the pectoral fin. Some short, rod-like bones might represent pectoral radials, but they have been displaced. The pectoral fin comprises at least eight unsegmented but distally bifurcating fin rays. More dorsal components of the shoulder girdle cannot be identified with confidence. A possible supracleithrum is visible as a triangular shaped bone in ventral view in V5785.

#### 4.2.8. Pelvic girdle and fin

The pelvic fin is located just posterior to the midpoint of the axial column. The pelvic girdle is not well preserved. The pelvic fin itself is substantially smaller than the pectoral fin and comprises at least four branched but unsegmented fin rays.

#### 4.2.9. Axial column and median fins

**Figure 3.**
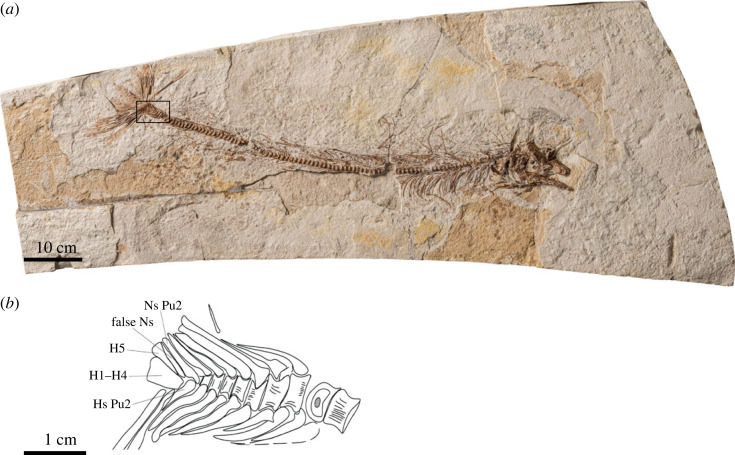
*Rhamphoichthys taxidiotis* gen. et sp. nov., WMNM P 64279. (*a*) Photograph of the entire specimen with the most complete post-cranial elements in right lateral view. (*b*) Interpretive line drawing of the region indicated by a black rectangle in (*a*). H, hypurals (numbered 1–5); Hs, haemal spine; Ns, neural spines; Pu, preural centrum (numbered 2).

No specimen preserves an intact vertebral column, with the most complete in P64279 (figure 3*a*). At least 26 centra are preserved between the back of the skull and a break in the fossil. This gap is broad enough to accommodate another two complete centra. Posterior to this, there are parts of 25 vertebrae before the first centrum bearing an obvious haemal arch and spine rather than ribs. On this basis, the number of abdominal vertebrae is constrained to no fewer than 56. From the first haemal-arch bearing vertebra to PU2, there are a total of 40 caudal vertebrae. The overall vertebral count is, therefore, in the region of 100.

Anterior centra are longer than they are deep and are thus spool-shaped rather than disc-shaped. However, the shape of vertebrae changes along the length of the column: anterior caudal vertebrae are roughly as long as deep, but those centra immediately preceding the caudal-fin endoskeleton are greatly foreshortened, being much shorter than they are deep. As far as can be seen, anterior and posterior surfaces of all vertebrae are deeply concave. The lateral surface of each centrum is covered with a fine network of intertwining ridges. Pairs of closely spaced, slit-shaped pits on the dorsal surfaces of vertebrae mark the articulations of neural arches. Subcircular pits apparent on the ventrolateral faces of abdominal vertebrae mark articulations of ribs. In V5785, several neural arches attach by gomphoses to their corresponding vertebrae. No abdominal haemal structures are visible. Similar pits mark the articulation of haemal arches in the caudal vertebrae but lie closer to the ventral midline of each vertebral centrum. Ribs are long, gently curved and bear a shallow longitudinal groove. They extend to the ventral margin of the body. Although some haemal and neural arches and spines are well preserved near the posterior of abdominal region and throughout the caudal region, they are generally not clearly distinguished from other rod-like ossifications in the anterior of the axial column. Haemal and neural spines are strongly reclined posteriorly, with the degree of deflection becoming more pronounced posteriorly. Throughout much of the caudal region, haemal and neural spines effectively extend horizontally, overlapping subsequent members of the series and giving the body a narrow profile. Numerous long, narrow intermuscular bones are present in the abdominal region. At least some more anterior intermusculars appear to attach to centra (epicentrals), while more posterior ones have more dorsal points of origin, on neural arches or spines (epineurals).

Pterygiophores are generally not well preserved in any specimen, but the distribution of fin rays provides some indication of the extent of the median fins. The dorsal fin commences almost immediately behind the skull and terminates shortly before the caudal fin. The overall extent of the anal fin is less clear, as rays are disrupted in the only specimen preserving this region largely intact. Individual fin rays, especially those of the dorsal fin, are very long. Some exceed the depth of the body, indicating a high dorsal fin. Anterior rays of the dorsal fin are particularly filamentous. Some fin rays do appear to bifurcate, but there is no evidence of segmentation.

#### 4.2.10. Caudal fin and supports

The bilobed caudal fin is large. Both examples are disrupted, so exact counts of rays are not possible. As in other fins, the lepidotrichia branch distally but do not segment. The bases of fin rays overlap much of the supporting endoskeleton. At least nine rays contributing to the dorsal lobe are preserved in P64279 (figure 3), with at least 10 in the lower lobe.

The centra immediately preceding the fin are very short, and their neural and haemal spines are thicker than those of more anterior vertebrae. As a consequence, adjacent neural and haemal spines in this region are closely applied. There is a large hypural plate (conventionally interpreted as the fusion of hypurals 1–4) and an autogenous dorsal hypural (conventionally interpreted as hypural 5). A sliver of bone applied to the anterodorsal margin of the autogenous hypural corresponds to the ossification identified by Taverne & Gayet [26] as a ‘false neural spine’ in other tselfatiiforms (see below). There is no obvious indication of a vestigial first preural centrum, but it could be obscured by other portions of the caudal skeleton. The second preural centrum bears an autogenous neural spine and haemal spine. The bases of their arches embrace the centrum to the degree that little of it is exposed laterally.

#### 4.2.11. Squamation

**Figure 4.**
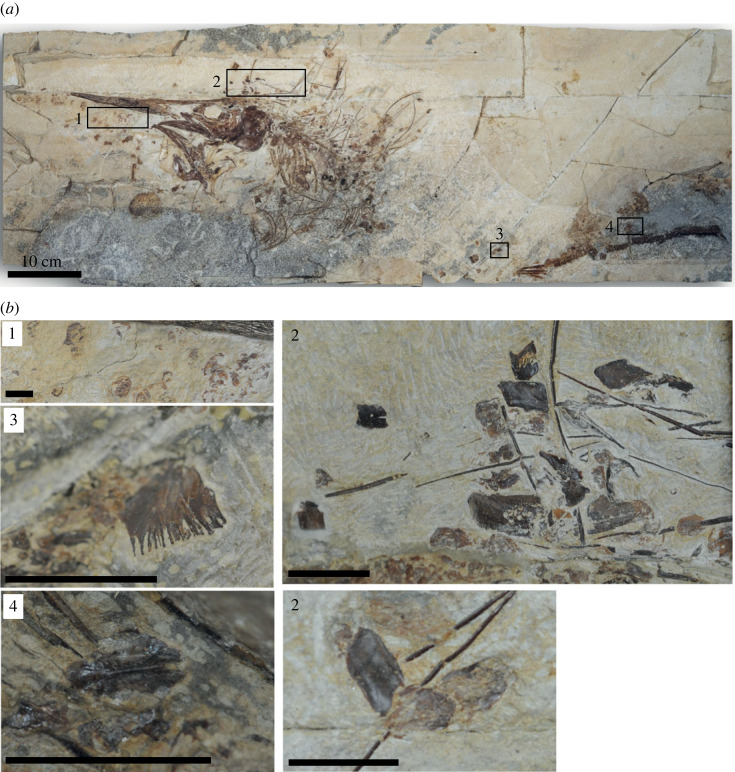
*Rhamphoichthys taxidiotis* gen. et sp. nov., holotype WMNM P 48342, Hesseltal Formation in the DIMAC Quarry, Halle, Westphalia. (*a*) Photograph of the entire specimen in left lateral view, showing the diversity of scale morphologies, indicated by the numbered frames (1–4). (*b*) Close-up photographs of (*a*): 1—ovoid and weakly mineralized scales; 2—rhombic and more robustly mineralized scales with few ovoid ones; 3—scale with a serrated posterior margin; 4—bilaterally symmetrical scales with a midline ridge and notch. Scale bars, 1 cm.

The scales of all specimens have been disrupted, although a few patches of articulated scales are preserved. There is a surprising diversity of scale morphologies (figure 4). The most common are ovoid, weakly mineralized scales (figure 4*b*-1), but these are joined by: rhombic and apparently more robustly mineralized scales (figure 4*b*-2); bilaterally symmetrical scales with a midline ridge and notch (figure 4*b*-4); and at least one scale with a serrated posterior margin (figure 4*b*-3). A substantially enlarged scale, or a series of imperceptibly overlapped scales, lie immediately posterior to the coracoids.

## 5. Discussion and conclusion

### 5.1. Systematic affinities

Taverne & Gayet [26] proposed eight synapomorphies for Tselfatiiformes, among which six are observed in *R. taxidiotis*: neural and haemal arches articulated by gomphosis on corresponding centra, compressed body, no anterior supramaxilla, absence of postcleithra, very long dorsal and anal fins, unsegmented dorsal- and anal-fin rays, and absence of epipleurals. The remaining two characters are either uncertain (the condition of the hypurals cannot be assessed due to extreme hypurostegy) or overprinted by probable apomorphies of this genus (the slender postcranium of *Rhamphoichthys* is unique among a group otherwise characterized by deep-bodied morphologies). The attribution of these specimens to Tselfatiiformes is, therefore, secure, even though the monophyly of the group is not yet confirmed (Cavin [36] versus Taverne & Gayet [26]).

Within the Tselfatiiformes, *R. taxidiotis* shows all the synapomorphies proposed by Taverne & Gayet [26] for Plethodidae: small teeth covering the jaws, palate and lingual plate; toothed bones pierced by numerous pits; presence of a complete osseous interorbital septum; antorbital, supraorbital and dermosphenotic articulated with one another along the lateral margin of the frontal; cleithrum bearing a long and obliquely orientated ventral arm and insertion of the pectoral fins high on the flank.

Taverne & Gayet [26] identified numerous features of plethodids excluding *Paranogmius*, the majority of which are found in the new genus: nasal, though not large, in articulation with frontal and mesethmoid; a single supramaxilla articulating with a deep notch in the maxilla; fusion of the angular and retroarticular, with the articular remaining autogenous; absence of supraneurals; dorsal fin extending from the back of the skull to near the caudal fin; epineurals restricted to abdominal region; presence of a neural spine on preural centrum 2 and absence of segmentation in dorsal- and anal-fin rays.

Plethodidae includes around 20 genera, and a detailed comparison to all these taxa is beyond the scope of this paper. Instead, we compare *R. taxidiotis* to the plethodids with relatively elongated rostra. Taverne & Gayet [26] recognized a clade comprising *Martinichthys*, *Pseudothryptodus*, *Thryptodus* and *Plethodus*, all united by two features apparent in our material: the premaxillae enlarged and fused together and the presence of a broad lingual tooth plate. However, within this group, and among all tselfatiiforms, *Martinichthys* (electronic supplementary material, figures S1 and S2) is the only genus with a greatly elongated rostrum. *Rhamphoichthys taxidiotis* shows even more extreme elongation, suggesting affinity to *Martinichthys*. *Rhamphoichthys taxidiotis* shares other autapomorphic characters with *Martinichthys*: the premaxillae joined dorsally and ventrally forming a secondary palate and hiding the vomer and the absence of the anterior articular condyle of the maxilla.

Taverne [25] synonymized many of the species of *Martinichthys* described by McClung [37], recognizing only two as valid: *M. brevis*, the type species (electronic supplementary material, figure S1), and *M. ziphioides* (electronic supplementary material, figure S2). These species differ in several features, but most obviously in proportions of the snout, where that of *M. ziphioides* is longer and thinner than that of *M. brevis*. However, *M. brevis* is more complete than the other species allowing more detailed comparison with *R. taxidiotis*.

Despite the common rostral elongation, the general morphology of *Rhamphoichthys* and *Martinichthys* rostra differs: the more modest rostrum of the latter is much thicker and more rounded compared to that of *R. taxidiotis*, where the rostrum is narrower and ends with a pointed to slightly rounded tip; the smooth rostrum of *R. taxidiotis* stands in contrast to the densely pitted examples in *Martinichthys* as well as other plethodids. Additionally, we note other differences in the skull. The cranial roof of *R. taxidiotis* is broad and its supraoccipital has a low crest, contrasting with a narrow roof and absence of a median crest in *Martinichthys*.

Other elements that differ between *M. brevis* and *Rhamphoichthys* are the hyomandibulae and the preopercles. The hyomandibula in *M. brevis* has a broad dorsal articular head and narrows down ventrally into a long and thick shaft according to Taverne's [25] description, whereas that in our material is hourglass shaped with a prominent constriction between its broad head and the thick shaft that becomes slightly larger ventrally after the constriction. Moreover, the preopercle of *M. brevis* displays a very short ventral branch and a longer but narrow dorsal branch. The preopercle of the new genus is crescent shaped with a large ventral limb. *Rhamphoichthys* differs from known species of *Martinichthys*, and indeed most other tselfatiiforms, in having abdominal vertebral centra that are elongate anteroposteriorly. This likely reflects the slender body of this genus relative to other members of the group. In addition to the double articular head of the quadrate not mentioned for any tselfatiiform.

### 5.2. Assignment of ‘*Protosphyraena*’ species to the newly erected genus

*Rhamphoichthys* aids in interpreting isolated Late Cretaceous rostra doubtfully assigned to the pachycormid *Protosphyraena*. Woodward [20] described several rostra of *Protosphyraena*, assigning them to different species based solely on the shape and ornamentation of the rostra. Mainwaring [38] questioned the assignment of some species to *Protosphyraena* or even to the pachycormids, two of which are *P. minor* (electronic supplementary material, figure S3) and *P. stebbingi* (electronic supplementary material, figure S4), from the English Chalk, that are relevant here. The latter author argued that these two species exhibit median sutures in their rostra indicating that paired bones contribute to the structure. This contrasts with the pachycormids, where the snout is composed exclusively of the median rostrodermethmoid. In addition, the flattened rostrum of these species does not agree with the more cylindrical rostra of *Protophyraena*.

Recent studies have re-evaluated the classification of ‘*P.*’ *stebbingi*, based on the material from the English Chalk and new material from northeastern Italy, assigning it to the Tselfatiiformes based on its rostrum composition and similarities to *Martinichthys* [23,39,40]. Similarly, ‘*P.*’ *minor* shares rostral characteristics with ‘*P.*’ *stebbingi*. Both species share with *R. taxidiotis* the following characteristics: rostra dorsoventral flattened, with an increasing width from tip to base, presence of a midline groove with raised edges, and similar surface ornamentation with parallel longitudinal and anastomosing ridges and stronger ridges, with different orientation on the lateral edges. Hence, we propose the inclusion of ‘*P.*’ *stebbingi* and ‘*P.*’ *minor* in the new genus *Rhamphoichthys.* However, due to the incompleteness of their specimens, composed of only isolated and fragmented rostra, it is suggested to regard them as *nomina nuda*.

### 5.3. *Rhamphoichthys* an ecological analogue for modern billfishes

The combination of an elongated rostrum and large body size exhibited by *Rhamphoichthys* is seen in several actinopterygians from Mesozoic and Cenozoic deposits [7,39,40], with some variations in the general morphology among taxa with this general morphotype (some differences include: relative lengths of upper and lower jaws; development of dentition; extension, size and number of dorsal and anal fins; placement of pectoral fins; presence or absence of pelvic fins). The similarity in morphology can be the result of convergent evolution, as these longirostrine fishes belong to distantly related groups, such as the pachycormids, tselfatiiforms and the billfishes. Past authors have suggested that rostra of ‘*P.*’ *stebbingi*, tselfatiiforms and *Protosphyraena* were suggested to be a convergent character associated with their feeding habits, similar to extant billfishes [40].

**Figure 5.**
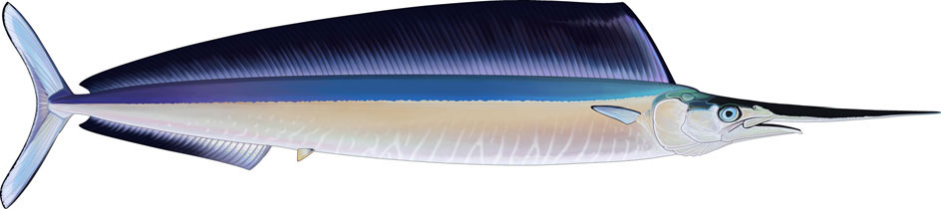
Reconstruction of *Rhamphoichthys taxidiotis* gen. et sp. nov. Artwork by Sky Jung.

Billfishes, a group of percomorph fishes appearing in the Early to Mid-Cenozoic, are characterized by their elongated premaxillae forming a non-protrusible rostrum or bill, bearing villiform teeth [7,41–43]. Molecular and anatomical analyses place billfishes within the Carangiformes, a group including a morphologically diverse assortment of mainly marine fishes (e.g. [44]). *Rhamphoichthys* (figure 5) shares a large, fusiform body shape with billfishes. Like extant billfishes, it has an upper jaw that is longer than the lower jaw. It also possesses a mixture of characters shared with both extant and extinct billfishes, such as high and long dorsal fin, a likely long anal fin as with the other plethodids, tiny teeth on the lower jaw, villiform teeth on their fused premaxillae and a bilobed caudal fin. However, it differs from billfishes in the high placement of the pectoral fin and large scales covering its body.

### 5.4. *Rhamphoichthys* a component of Cretaceous marine ecosystems

**Figure 6.**
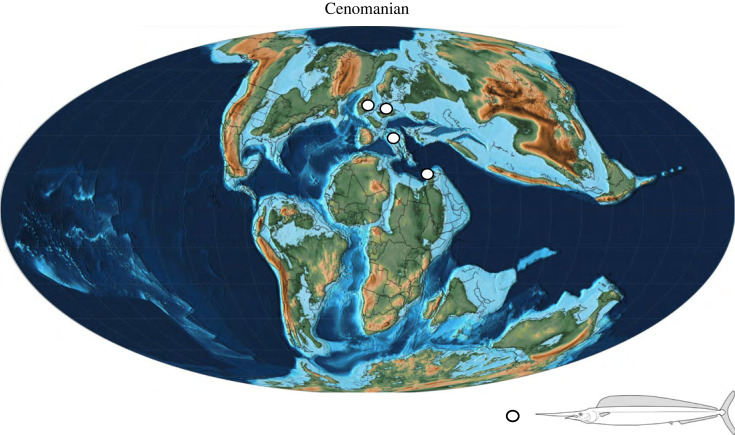
Palaeomap of the Cenomanian (96.6 Ma; adapted from Scotese [45]), showing the palaeogeographic distribution of *Rhamphoichthys taxidiotis* gen. et sp. nov. (circle) during this period.

Although details of its overall morphology are new, *Rhamphoichthys* does not appear to be exceptionally rare in Cretaceous marine settings. The inclusion of ‘*P.*’ *stebbingi* and ‘*P.*’ *minor*, as *Rhamphoichtys* sp., makes it a relatively common fish roaming the central Tethys and the Boreal realms during the Cenomanian with representatives from Germany, Italy, Lebanon and the English Chalk, UK (figure 6).

Numerous specimens from these localities provide evidence of its abundance [20,22,23,39,40,46]. Two specimens, from the Cenomanian Lebanese deposits, can be potentially added to this genus: a complete undescribed specimen mentioned by Gayet *et al.* [24, p.168] exhibiting an elongated rostrum and fusiform large body, noted by Amalfitano *et al.* [40] as a possible ‘*P.*’ *stebbingi*; and a specimen from the MNHN (HDJ-250) (electronic supplementary material, figure S5) consisting of only two frontals identical to those of *R. taxidiotis* (figure 2*d*); however, due to its incomplete nature, no formal assignment can be made.

## Data Availability

New data can be found in either the paper or electronic supplementary material, figures [47].
